# Burden and etiology of moderate and severe diarrhea in children less than 5 years of age living in north and south of China: Prospective, population-based surveillance

**DOI:** 10.1186/s13099-021-00428-2

**Published:** 2021-05-24

**Authors:** Hong-Lu Zhou, Theresa Bessey, Song-Mei Wang, Zhao-Jun Mo, Leslie Barclay, Jin-Xia Wang, Can-Jing Zhang, Jing-Chen Ma, Chao Qiu, Gan Zhao, Rong-Cheng Li, Yu-Liang Zhao, Baoming Jiang, Xuan-Yi Wang

**Affiliations:** 1 Key Laboratory of Medical Molecular Virology of Ministry of Education & Ministry of Health, and Institutes of Biomedical Sciences, Fudan University, Shanghai, 200032 People’s Republic of China; 2 Viral Gastroenteritis Branch, Division of Viral Diseases, National Center for Immunization and Respiratory Diseases, Centers for Disease Control and Prevention, Atlanta, GA USA; 3 Laboratory of Molecular Biology, Training Center of Medical Experiments, School of Basic Medical Sciences, Fudan University, Shanghai, 200032 People’s Republic of China; 4 Hebei Province Center for Disease Control and Prevention, Shijiazhuang, 050021 People’s Republic of China; 5 Guangxi Center for Disease Control and Prevention, Nanning, 530028 People’s Republic of China; 6 Children’s Hospital, Fudan University, Shanghai, 201102 People’s Republic of China

**Keywords:** Burden, Etiology, Children, Diarrhea, China

## Abstract

**Background:**

Diarrhea remains the leading cause of childhood illness in China. Better understanding of burden and etiology of diarrheal diseases is important for development of effective prevention measures.

**Methods:**

Population-based diarrhea surveillance was conducted in Sanjiang (southern China) year-round and Zhengding (northern China) in autumn/winter. Stool specimens were collected from children < 5 years of age experiencing diarrhea. The TaqMan Array Card (TAC), based on multiplex real-time PCR, was applied to detect multiple enteric microbial agents simultaneously. Results using these methods were compared to those derived from conventional PCR assays.

**Results:**

During the study period, 6,380 children in Zhengding and 3,581 children in Sanjiang < 5 years of age participated. Three hundred and forty (31.2%) and 279 (22.9%) diarrhea episodes were identified as moderate-to-severe in the two counties, with incidence of 60.4 and 88.3 cases per 1,000 child-years in Zhengding and Sanjiang, respectively. The five most frequently detected bacterial and viral agents in Sanjiang were adenovirus, enterovirus, enteroaggregative *Escherichia coli* (EAEC), rotavirus, and sapovirus all the year round, while the most common viral agents in Zhengding were rotavirus, followed by astrovirus and adenovirus during the cool season. Compared to conventional PCR assay, the average incremental detection via the TAC method was twofold.

**Conclusion:**

Our study demonstrated high diversity and prevalence of multiple major bacterial and viral agents, including rotavirus and calicivirus, among children in China. Further studies are needed to define the public health significance of neglected but frequently detected pathogens such as EAEC, enterotoxigenic *E. coli*, *Campylobacter*, adenovirus, and enterovirus.

**Supplementary Information:**

The online version contains supplementary material available at 10.1186/s13099-021-00428-2.

## Key Points

Currently bacterial and viral agents in the gastrointestinal tract are under-detected and poorly defined in developing countries. A new sensitive TAC assay provided comprehensive identification of microorganisms, including neglected pathogens, in Chinese children with moderate and severe diarrhea.

**Supplementary Information:**

The online version contains supplementary material available at 10.1186/s13099-021-00428-2.

## Background

Globally, despite significant advances in fighting childhood diarrhea through sanitation improvement, safe water supplies, and vaccine implementation during the past decades, diarrheal diseases remained the fifth cause of death for children < 5 years of age in 2016 [1]. The World Health Organization and the United Nations Children’s Fund (UNICEF) launched a global action plan in 2013, aimed to end preventable childhood deaths due to pneumonia and diarrhea by 2025 [2]. However, comprehensive understanding of diarrhea etiology remains a significant gap between the action plan and the real-world situation. A large percentage of diarrhea cases are reported without any etiological diagnosis.

According to China’s National Notifiable Infectious Disease Reporting system (NIDR), 1,275,290 cases of infectious diarrhea were reported in 2017 (cholera, dysentery, and enteric fever were excluded in this total), resulting in an incidence of 92.2 per 100,000 person-years [3]. Of these, few (~ 9.0%) cases were laboratory confirmed [4]. Moreover, considering the likelihood of underreporting via the NIDR system, the true disease burden of infectious diarrhea may be several times greater than reported. Therefore, better understanding of burden and etiology of diarrheal diseases is important for development of preventive strategies, such as routine immunizations.

Population-based diarrhea surveillance in children under age 5 was conducted in 2 rural counties: Zhengding county of Hebei province (north) and Sanjiang county of Guangxi province (south) in China in 2012 and 2013. The occurrence of rotavirus, noroviruses, sapoviruses, astroviruses, and enteric adenoviruses were analyzed. However, only a low percent (~ 25%) of diarrhea cases contained one of abovementioned 4 viruses during the 1-year surveillance period using PCR methods [5]. Even if the focus was on the epidemic season, no viral agents were detected in half of diarrhea episodes. Currently, the cause for most diarrhea episodes in Chinese children remains unknown. To address this knowledge gap, a multipathogen TaqMan Array Card (TAC) [6] was employed to investigate etiologies in children with moderate-to-severe diarrhea (MSD), utilizing specimens collected from the abovementioned population-based surveillance effort.

## Methods

### Study population

As described elsewhere, in Sanjiang County, 3,613 children were monitored between January 1 and December 31, 2013. In Zhengding County, 6,443 children were followed from October 1, 2011 to March 31, 2012 [5]. Children who presented to a participating health care facility (including village clinics, township hospitals and county hospitals) with acute diarrhea and whose parents or guardians provided informed consent were enrolled. Information on demographics, medical history, physical examination, and the management plan during the full course of illness, as well as a stool specimen at presentation were collected and stored at − 20 °C freezer. After assessing severity of illness for each patient based on a modified Vesikari scoring system [7], fecal specimens from children who presented with MSD were selected for TAC testing. This study was reviewed and approved by the Institutional Review Board (IRB) of the Institutes of Biomedical Sciences, Fudan University. Written informed consent was obtained from the parent/guardian of each child. This research did not require review by the institutional review board of Centers for Disease Control and Prevention (CDC) because the CDC tested preexisting, anonymous specimens.

### Conventional PCR assay

All stool samples were tested for rotavirus, norovirus, sapovirus, astrovirus, and adenovirus using conventional PCR assays in the laboratory of Fudan university [5]. To identify the genotype of viruses, all PCR products were purified and sequenced using Sanger dideoxy termination sequencing (Biosune Co., Ltd., Shanghai).

### Multipathogen TaqMan array card assay

#### Nucleic acid extraction

Since surveillance in Sanjiang was year-round, covering the peak(s) of seasonal diarrhea resulting from bacterial and viral infections, total nucleic acid (TNA) of bacteria and viruses were extracted using MagMAX™ Total Nucleic Acid Isolation Kit (Thermo Fisher Scientific, Waltham, MA, USA) on KingFisher Flex (Thermo Fisher Scientific, Waltham, MA, USA). As an extrinsic control, bacteriophage MS2 (ATCC, Manassas, VA, USA) was spiked in lysis buffer to monitor the efficiency or inhibition of extraction and amplification of each sample. While surveillance in Zhengding only focused on viral diarrhea, TNA of viruses were extracted using the Tianlong virus RNA/DNA extraction kit on the NP968 3S system (Tianlong, Xi’an, China). Procedures were performed according to the manufacturer’s instructions unless otherwise stated. All isolated TNA were stored at − 80 °C prior to assays.

#### TAC design and procedure

The panel of TAC (Applied BiosystemsTM, USA) in US CDC used in this study included 6 viral, 13 bacterial, and 5 parasitic agents as previously described (Additional file 1: Table S1) [6]. Any quantification cycle (Cq) result of < 45 was considered positive.

The Luminex xTAG gastrointestinal pathogen panel assay (GPP) (Luminex Molecular Diagnostics, Austin, TX, USA) as a qualitative multiplex test has been shown to be highly sensitive and specific for a variety of pathogens in clinical settings globally [8–10]. In this study, the xTAG GPP platform in US CDC was used to evaluate the results of rotavirus A, norovirus GI/GII, and adenovirus 40/41 from TAC assays.

#### Statistical analysis

We used SAS (SAS Institute Inc., Cary, NC, USA) for statistical analysis. For binary data, the Chi square test was used, or Fisher’s exact test when data were sparse. For comparison of Ct values, the Mann–Whitney U test was used. For validation of the TAC assay, sensitivity, specificity, and kappa coefficient with 95% confidence intervals (95% CI) were calculated using GPP test results as references. The incidence rate of viral diarrhea was calculated as the number of diarrheal illness episodes with stool specimens testing positive for rotaviruses, noroviruses, sapoviruses, adenoviruses, and astroviruses among the surveillance participants per 1,000 child-years. The proportions of cases positive for each agent were calculated. Ct values were presented as the mean of two duplicate wells for specimens yielding positive results. If these two duplicate wells presented discordant results (one was positive via Ct value and one was negative), “indeterminate” comments were assigned to the wells; these wells were not included in subsequent calculations. All P values were two-tailed with < 0.05 considered statistically significant.

## Results

### Summary of the study population and incidence of MSD

During the study period, 6,380 children in Zhengding and 3,581 children in Sanjiang under 5 years of age participated, and experienced 1,211 and 1,258 diarrhea episodes respectively. The overall incidence of diarrhea was 215.0 and 398.2 episodes per 1000 child-years in Zhengding and Sanjiang. Of these episodes, 340 (31.2%) and 279 (22.9%) episodes were identified as moderate-to-severe diarrhea with incidence of 60.4 and 88.3 per 1000 child-years in Zhengding and Sanjiang, respectively (Tables 1 and 2). As enterococcus is a common commensal organism in the human intestine, not considered a pathogen associated with diarrhea [11], it was not included in subsequent analyses despite a high detection rate (69.2%) and incidence (61.1 per 1000 child-years) in Sanjiang.

**Table 1:** Incidence (per 1000 child-years) of total MSD, MSD with any agent, and MSD with a specific agent, with 95% confidence interval, by age stratum in Sanjiang

Sanjiang	0–11 months	12–23 months	24–59 months	Total
MSD-total	134.0 (106.3–161.7)	130.4 (103.9–156.9)	61.3 (50.7–71.9)	88.3 (78.4–98.2)
MSD-agent	113.4 (87.6–139.2)	117.6 (92.3–142.9)	54.6 (44.5–64.7)	77.9 (68.6–87.2)
1	Enterovirus	Adenovirus	Enterovirus	Adenovirus
	53.3 (35.1–71.5)	56.4 (38.3–74.5)	26.0 (19.0–33.0)	34.5 (28.1–40.9)
2	Adenovirus	EAEC	Adenovirus	Enterovirus
	43.0 (26.5–59.5)	49.9 (32.8–67.0)	25.0 (18.1–31.9)	34.2 (27.9–40.5)
3	EAEC	Enterovirus	EAEC	EAEC
	37.8 (22.3–53.3)	41.9 (26.1–57.7)	18.9 (12.9–24.9)	28.5 (22.7–34.3)
4	Rotavirus	Rotavirus	Rotavirus	Rotavirus
	36.1 (20.9–51.3)	37.0 (22.2–51.8)	12.8 (7.8–17.8)	21.8 (16.7–26.9)
5	Norovirus	Astrovirus	Sapovirus	Sapovirus
	25.8 (12.9–38.7)	29.0 (15.8–42.2)	11.7 (6.9–16.5)	15.2 (10.9–19.5)
6	Sapovirus	Norovirus	Adenovirus 40/41	Adenovirus 40/41
	22.3 (10.3–34.3)	27.4 (14.6–40.2)	11.2 (6.5–15.9)	14.6 (10.4–18.8)
7	ETEC	Adenovirus 40/41	ETEC	Norovirus
	18.9 (7.8–30.0)	25.8 (13.3–38.3)	10.2 (5.7–14.7)	14.6 (10.4–18.8)
8	Adenovirus 40/41	ETEC	Astrovirus	ETEC
	13.7 (4.3–23.1)	22.5 (10.8–34.2)	7.2 (3.5–10.9)	14.2 (10.1–18.3)
9	Astrovirus	*Campylobacter*	Norovirus	Astrovirus
	13.7 (4.3–23.1)	22.5 (10.8–34.2)	7.2 (3.5–10.9)	12.7 (8.8–16.6)
10	*Campylobacter*	Sapovirus	*Ascaris lumbricoides*	*Campylobacter*
	13.7 (4.3–23.1)	19.3 (8.5–30.1)	6.1 (2.7–9.5)	10.4 (6.9–13.9)

*EAEC*  enteroaggregative *E coli*, *ETEC*  enterotoxigenic *E coli*

**Table 2:** Incidence (per 1000 child-years) of total MSD, MSD with any agent, and MSD with a specific agent, with 95% confidence interval, by age stratum in Zhengding

Zhengding	0–11 months	12–23 months	24–59 months	Total
MSD-total	138.5 (119.0–158.0)	102.4 (85.7–119.1)	13.6 (9.6–17.6)	60.4 (54.2–66.6)
MSD-agent	125.2 (106.5–143.9)	100.0 (83.5–116.5)	11.1 (7.4–14.8)	55.6 (49.6–61.6)
1	Rotavirus	Rotavirus	Rotavirus	Rotavirus
	77.9 (62.8–93.0)	72.4 (58.1–86.7)	7.0 (4.1–9.9)	36.9 (32.0–41.8)
2	Astrovirus	Astrovirus	Adenovirus 40/41	Astrovirus
	48.9 (36.7–61.1)	44.1 (32.8–55.4)	5.4 (2.8–8.0)	22.5 (18.6–26.4)
3	Adenovirus	Adenovirus	Sapovirus	Adenovirus
	47.3 (35.3–59.3)	41.7 (30.7–52.7)	5.4 (2.8–8.0)	22.4 (18.5–26.3)
4	Adenovirus 40/41	Adenovirus 40/41	Adenovirus	Adenovirus 40/41
	37.3 (26.6–48.0)	35.4 (25.2–45.6)	5.1 (2.6–7.6)	19.0 (15.4–22.6)
5	Norovirus	Sapovirus	Astrovirus	Sapovirus
	34.8 (24.5–45.1)	32.3 (22.6–42.0)	3.8 (1.7–5.9)	16.5 (13.2–19.8)
6	Sapovirus	Norovirus	Enterovirus	Norovirus
	29.0 (19.5–38.5)	24.4 (15.9–32.9)	1.9 (0.4–3.4)	13.8 (10.8–16.8)
7	Enterovirus	Enterovirus	Norovirus	Enterovirus
	22.4 (14.0–30.8)	17.3 (10.1–24.5)	1.6 (0.2–3.0)	9.8 (7.2–12.4)

## Etiological and epidemiological characteristics of MSD in Sanjiang

**Fig. 1:**
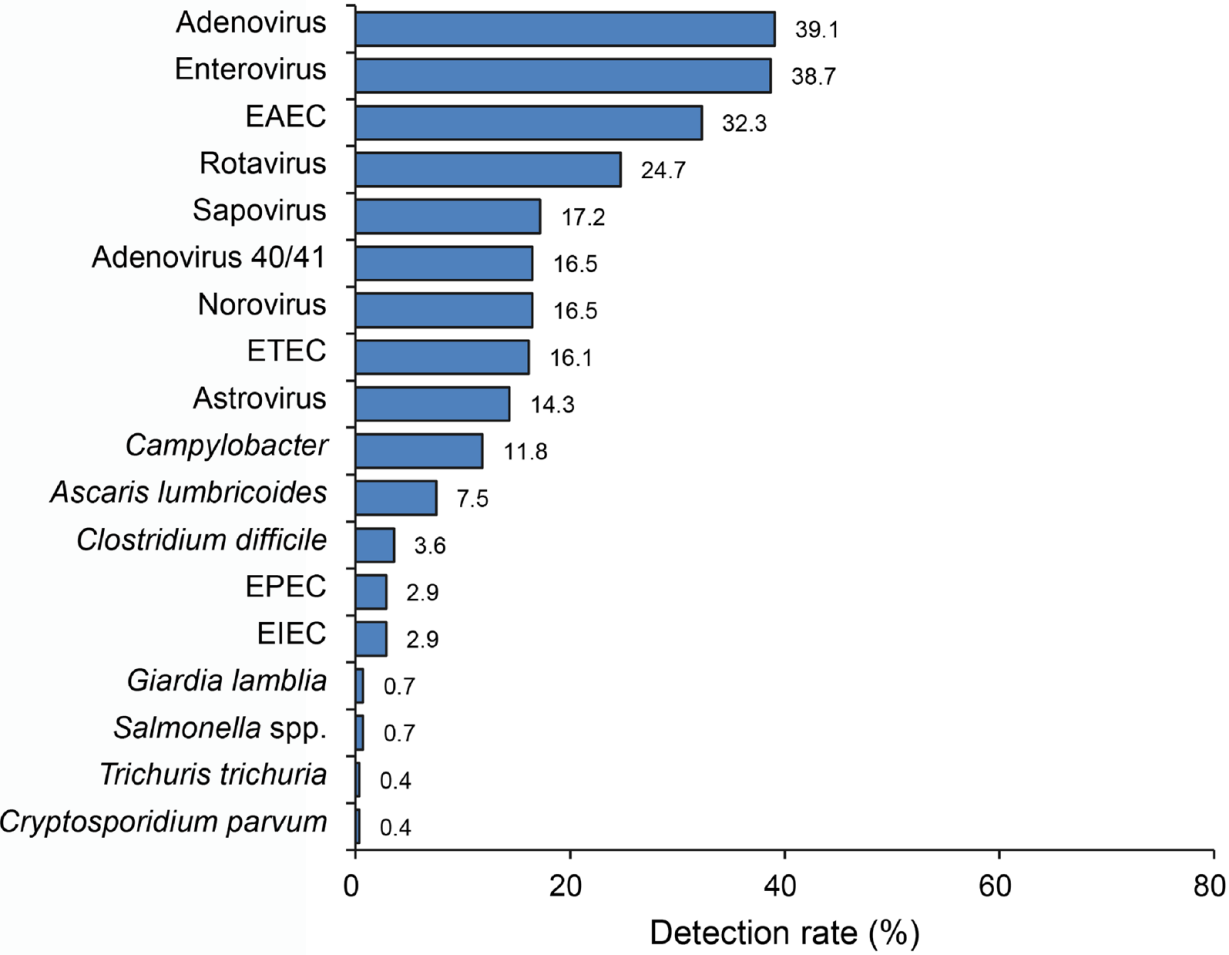
The profile of microbial agents detected in children with MSD in Sanjiang, Guangxi *EAEC *enteroaggregative *E coli*. *ETEC* enterotoxigenic *E coli*.* EIEC*  enteroinvasive *E coli*. *EPEC*  enteropathogenic *E coli*. Adenovirus: all adenovirus serotypes except 40 and 41

In Sanjiang, during year-round surveillance covering peaks of both bacterial and viral infections, the most frequently detected (detection rate > 10%) agents in children with MSD were adenovirus (39.1%), enterovirus (38.7%), Enteroaggregative *E. coli* (EAEC) (32.3%), rotavirus (24.7%), sapovirus (17.2%), enteric adenovirus 40/41 (16.5%), norovirus (16.5%), Enterotoxigenic *E. coli* (ETEC) (16.1%), astrovirus (14.3%) and *Campylobacter* (11.8%) (Fig. 1), resulting in incidence of 34.5, 34.2, 28.5, 21.8, 15.2, 14.6, 14.6, 14.2, 12.7 and 10.4 per 1,000 child-years, respectively (Table 1).

**Fig. 2:**
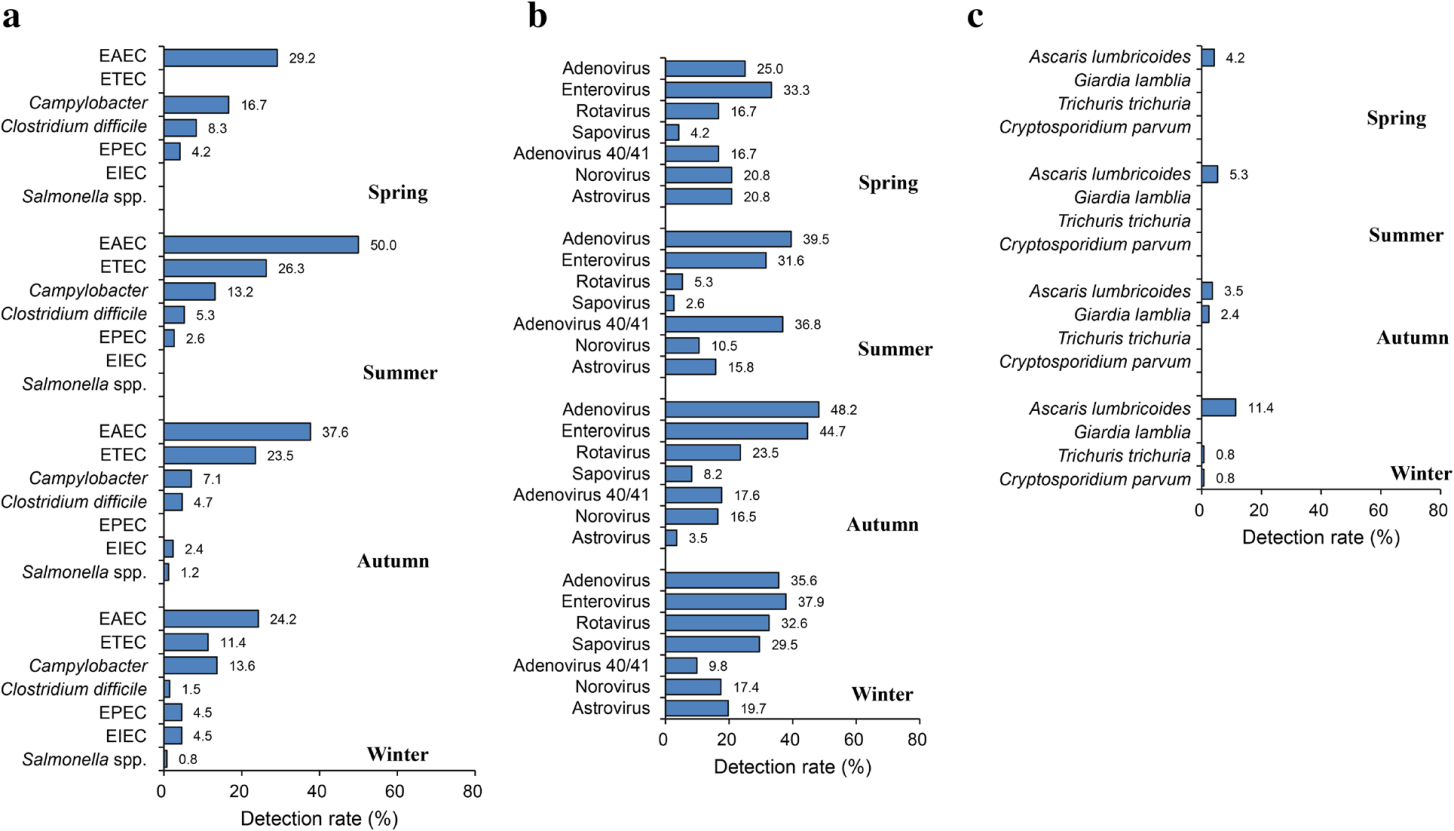
The profile of microbial agents detected in children with MSD by season in Sanjiang. **a** bacterial agents. **b** viral agents. c. parasitic agents. Spring: Mar to May; Summer: Jun to Aug; Autumn: Sept to NoV; Winter: Dec to Feb

The prevalence of microbial agents in Sanjiang was examined by season (Fig. 2). For bacterial infections, EAEC and ETEC were more frequently detected during summer (50.0%, and 26.3%), while *Campylobacter* and *C. difficile* appeared in spring (16.7%, and 8.3%). By contrast, all viral agents were detected throughout the year, except summer when adenovirus 40/41 peaked sharply and the rotavirus detection rate was low. For parasitic infections, only *A. lumbricoides* was detected frequently and year-round, though a winter peak was observed.

**Fig. 3:**
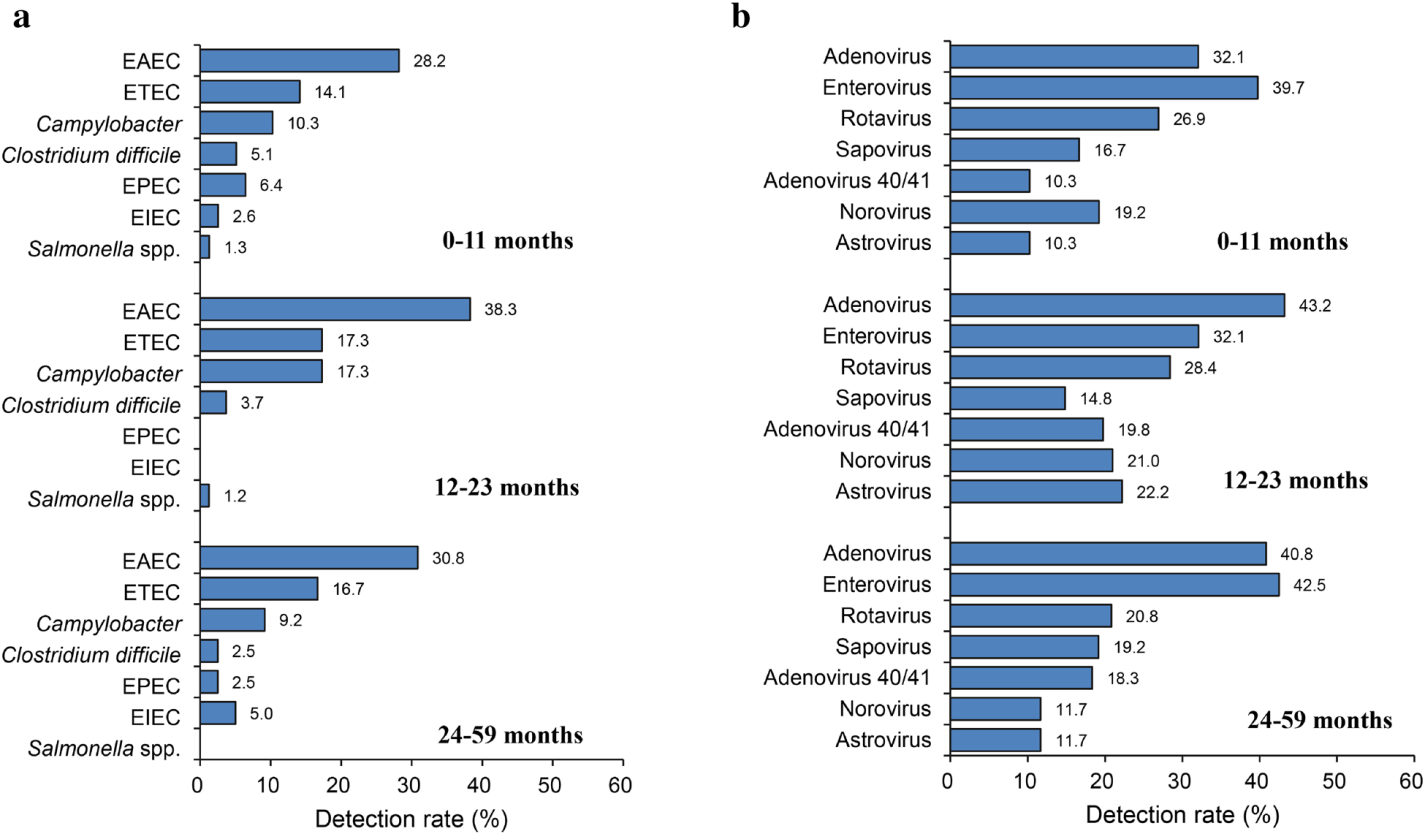
The profile of microbial agents detected in children with MSD by age stratum in Sanjiang. **a** bacterial agents. **b** viral agents

No significant differences among tested agents were observed in the age distribution of children infected (Fig. 3). Comparatively, the bacterial agents, represented by EAEC, ETEC, and *Campylobacter* were detected more frequently in children aged 12–59 months. More specifically, for bacterial agents with a detection rate > 10% (EAEC, ETEC, and *Campylobacter*), the highest incidence was found in children aged 12–23 months (49.9, 22.5, 22.5 per 1,000 child-years), and followed by incidence in children aged 0–11 months (37.8, 18.9, 13.7 per 1,000 child-years). For viral agents with a detection rate > 10%, adenovirus, rotavirus, adenovirus 40/41, norovirus, and astrovirus infected children aged 12–23 months (56.4, 37.0, 25.8, 27.4 and 29.0 per 1,000 child-years respectively) most frequently, followed by children aged 0–11 months (43.0, 36.1, 13.7, 25.8 and 13.7 per 1,000 child-years respectively). Conversely, the highest incidences of enterovirus and sapovirus were found in children aged 0–11 months (53.3 and 22.3 per 1,000 child-years), followed by that in children aged 12–23 months (41.9 and 19.3 per 1,000 child-years) (Table 1).

### Comparison on etiological and epidemiological characteristics of viral infection at peak season between Sanjiang and Zhengding

**Fig. 4:**
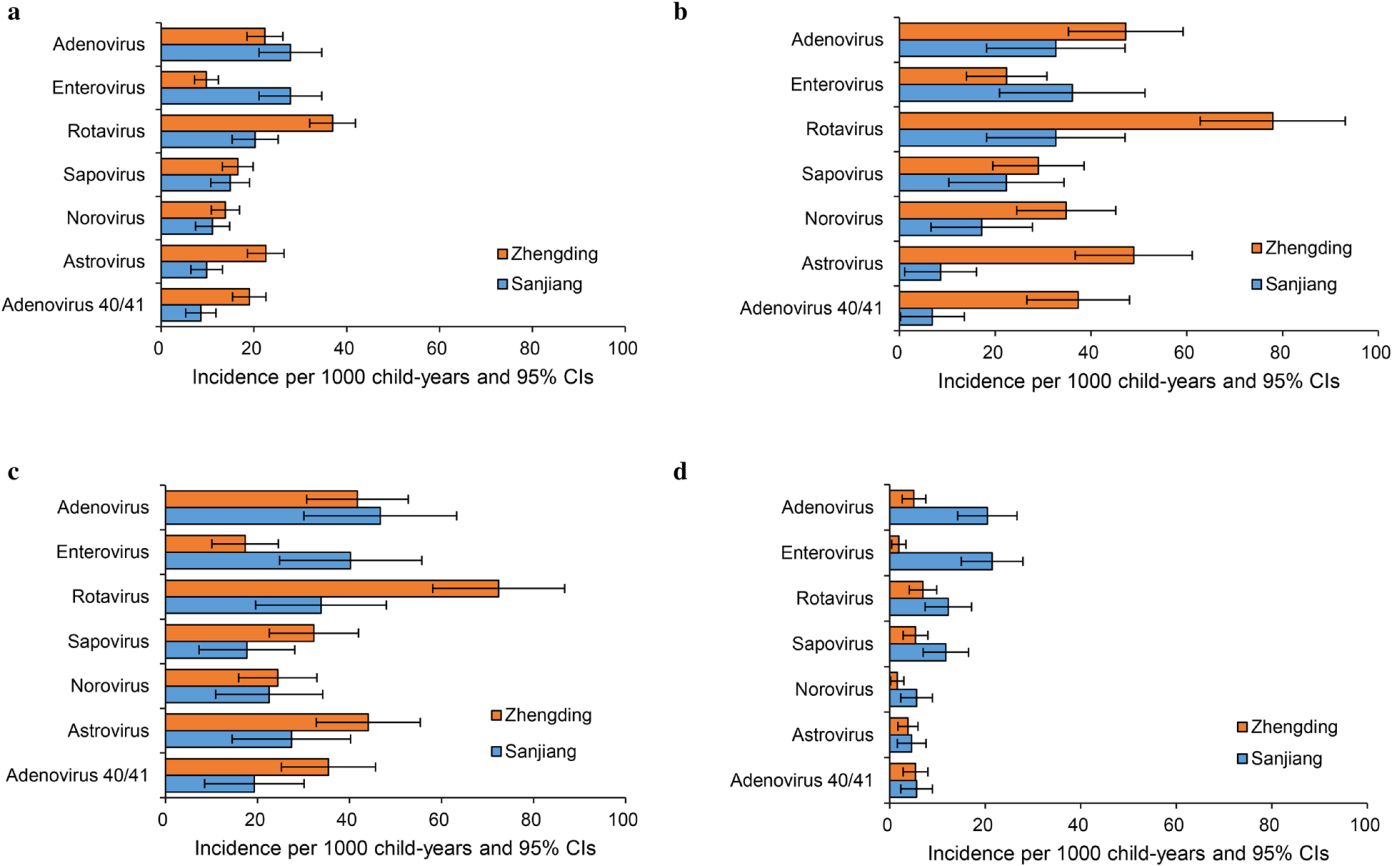
Viral agent-specific incidence (per 1000 child-years) of MSD by age stratum in Sanjiang and Zhengding from October to March. **a** total children aged 0–59 months **b** children aged 0–11 months. **c** children aged 12–23 months. **d** children aged 24–59 month. Adenovirus: all adenovirus serotypes except 40 and 41

Since we only collected fecal specimens from children with MSD in Zhengding from October through March when viral agents like rotavirus usually peaked each year, we compared virus-specific incidence profiles between Zhengding and Sanjiang in the same time frame (Fig. 4). Of these viral agents, Zhengding had higher incidence of rotavirus (36.9 per 1,000 child-years), astrovirus (22.5 per 1,000 child-years), and adenovirus 40/41 (19.0 per 1,000 child-years) than Sanjiang. Conversely, Sanjiang had higher incidence of enterovirus (27.9 per 1,000 child-years) than Zhengding. Both places had high incidence of adenovirus (22.4–27.9 per 1,000 child-years) (Fig. 4a).

After age stratification, increased burdens due to viral pathogens were measured in children aged 0–11 months and 12–23 months (Fig. 4b, c); incidence decreased significantly in children aged 24–59 months (Fig. 4d). It was particularly noteworthy that rotavirus, norovirus, astrovirus, and adenovirus 40/41 caused a contrasting incidence of MSD in children aged 0–11 months in Zhengding in comparison with that in Sanjiang; incidence was 77.9, 34.8, 48.9 and 37.3 per 1,000 child-years, respectively, accounting for 45.2%, 53.8%, 46.5% and 42.1% of all cases in children < 5 years of age.

### Comparison between GPP assay and TAC assay

In order to validate the TAC results, diagnostic testing using GPP test results as reference was performed (Table 3). Of 265 specimens tested, the overall sensitivity and specificity for all three viruses of TAC was 89.5% and 92.7% respectively, with individual virus assay sensitivities ranging from 77.0% to 95.7% and specificities from 83.5% to 96.6%. The TAC assays for norovirus GI/GII exhibited the lowest sensitivity, ranging from 68.1% to 85.9%, whereas the assays for rotavirus A had the lowest specificity, ranging from 77.0% to 89.9%. Overall, though, the kappa coefficient for each virus was greater than 0.75, indicating substantial consistency between TAC and GPP results.

**Table 3:** Comparison of GPP and TAC assay results

Pathogen	GPP	TAC		Total	Sensitivity %(95% CI)	Specificity %(95% CI)	Kappa(95% CI)
		positive	negative				
Rotavirus A	Positive	132	6	138	95.7 (92.3–99.1)	83.5 (77.0–89.9)	0.79 (0.72–0.87)
	Negative	21	106	127			
	Total	153	112	265			
Norovirus GI/GII	Positive	67	20	87	77.0 (68.1–85.9)	96.6 (94.0–99.3)	0.77 (0.68–0.85)
	Negative	6	172	178			
	Total	73	192	265			
Adenovirus 40/41	Positive	101	8	109	92.7 (87.8–97.6)	95.5 (92.3–98.8)	0.88 (0.83–0.94)
	Negative	7	149	156			
	Total	108	157	265			

Sensitivity and specificity of TAC assay calculated using GPP test results as reference

### Comparison between conventional PCR assay and TAC assay

**Fig. 5:**
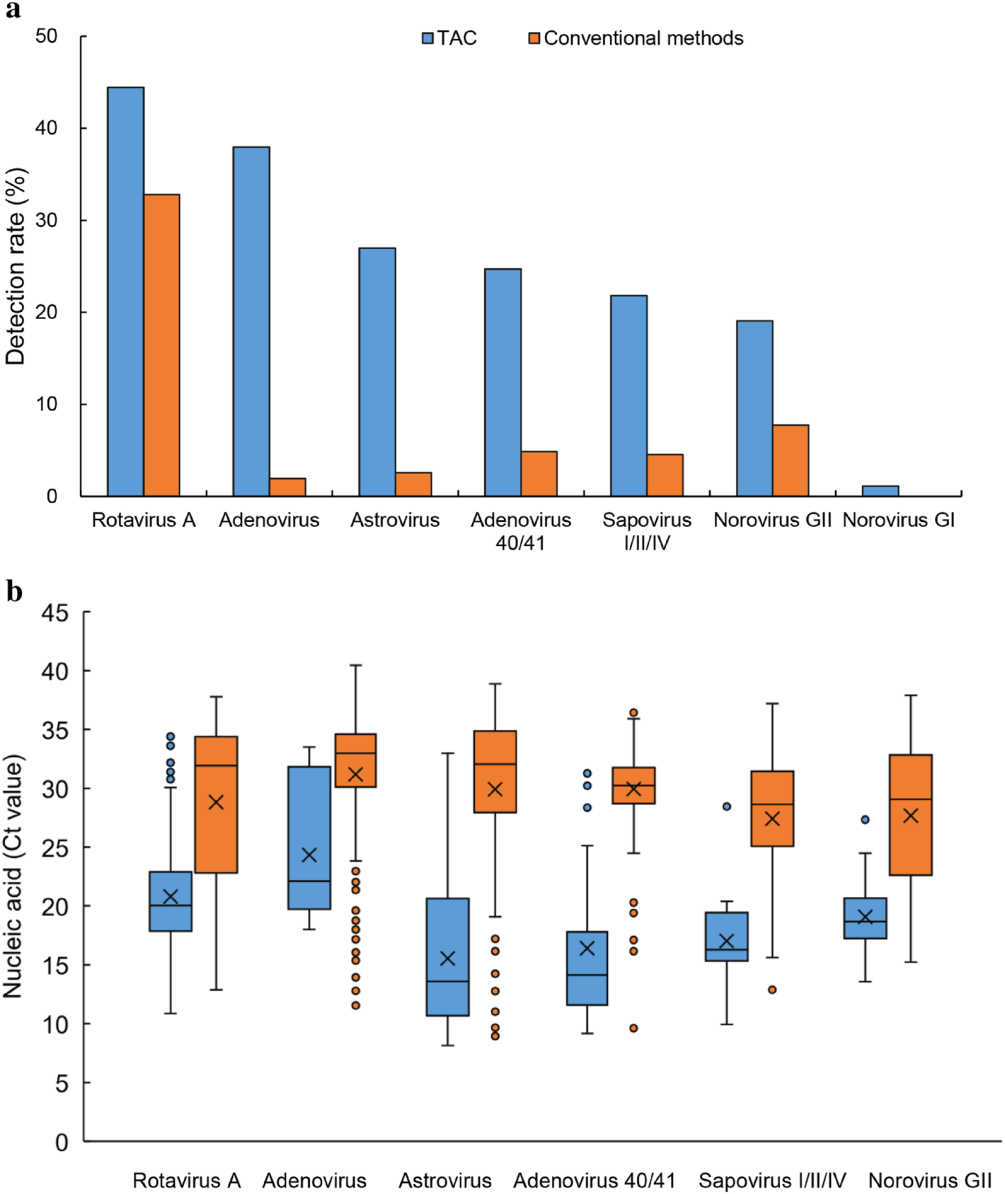
Comparison of the viral detection rates between TAC and conventional PCR assays. **a** Pathogen-specific detection rate between TAC and conventional PCR assay. **B** TAC Ct values in conventional PCR-positive and -negative samples. PCR positive: blue box; PCR negative: orange box; significant difference was detected among PCR-positive and -negative samples for all viral pathogens. Adenovirus: all adenovirus serotypes except 40 and 41

Compared to conventional PCR assay, TAC displayed more sensitivity—an average increment of twofold. However, the increase in sensitivity varied among different viruses. The highest increase was found with adenovirus detection, which was 18.6-fold, followed by astrovirus (9.4-fold) and adenovirus 40/41 (4.1-fold). The lowest increase was found with rotavirus detection; the difference between the two assay methods was only 0.4-fold (Fig. 5a). Ct values derived from TAC assay were grouped by conventional PCR result for each viral agent. Overall, the Ct values of conventional PCR-negative samples were significantly higher than those of PCR-positive samples for all five viruses (*p* < 0.001). Out of these five viral agents, the average Ct values between PCR-negative and -positive samples in astrovirus, adenovirus 40/41, and sapovirus were notably more discrete (Fig. 5b).

## Discussion

We previously reported that viral diarrhea, especially that caused by rotavirus and norovirus in Chinese children < 5 years, remained an unmet public health concern with a significant disease burden [5, 12]. Even so, our estimates were likely conservative, due to the limits of the laboratory methods applied, since viral agents were detected in 52.5% of samples [5]. Recently, TAC platforms detecting multiple pathogens simultaneously were developed for surveillance of respiratory and enteric infectious disease with heightened assay efficiency and sensitivity [6, 13–17].

When applying the TAC assay to reanalyze enteric pathogens in samples derived from our previous population-based surveillance, a significant increase of detection rates was observed compared to the conventional PCR assays. In particular, a ~ 30% increase was observed in detection of adenovirus (40/41 excluded) and astrovirus. Further analysis indicated that the increases resulted from detection in specimens containing lower quantities of microbial nucleic acids. Based on the TAC assay, revised incidence of rotavirus infection in children with MSD was 36.9 and 21.8 cases per 1,000 child-years in Zhengding and Sanjiang, respectively, followed by norovirus (13.8 vs 14.6 cases per 1,000 child-years in Zhengding and Sanjiang, respectively). These estimates were 0.4–1.5 fold higher than those derived from conventional PCR assays. This will undoubtedly further enhance demand for rotavirus and norovirus vaccines for disease control [5]. At the same time, a diagnostic test using GPP as reference further validated the accuracy of TAC results—valuable evidence for future applications.

Surprisingly, after adjusting the sensitivity of laboratory assay, whether in Sanjiang (South China) or Zhengding (North China), the detection rate of adenovirus infections was consistently elevated, and incidence even exceeded that of norovirus and rotavirus infections in Sanjiang. In addition, high rates of enterovirus were observed in Sanjiang. Classically, adenovirus 40/41 and type 52 are recognized as the causative agents of gastroenteritis, while other types contributed to an array of clinical disease including conjunctivitis, hepatitis, myocarditis, and pneumonia [18]. Enterovirus is generally more likely to lead to respiratory symptoms and neurologic diseases [19]. Thus, the clinical and public health significance of such infections in Chinese children need to be further studied. More frequent detection of enterovirus in Sanjiang than in Zhengding might be attributed to higher tropical temperatures. Enterovirus appears to occur year-round with higher prevalence in late spring throughout early autumn [20]. Besides, the high rate of enterovirus detection might be due to the administration of live oral poliovirus vaccine (OPV) that caused increased enterovirus detected in stool samples [21].

Though it is the most populous country in the world, China is still a developing country, with unbalanced economic conditions across the country. Generally speaking, Sanjiang and Zhengding are less developed with relatively lower per capita gross domestic product [22]. China has benefitted from a booming economy over the past two decades, and the spectrum of pathogens causing diarrhea has changed significantly. Viral infections typified by rotaviruses, noroviruses, and sapoviruses have become major pathogens, rather than bacterial infections [5, 12]. Although more bacterial targets were included in the TAC assay, in Sanjiang, the top five agents detected in children with MSD were adenovirus, enterovirus, EAEC, rotavirus and sapovirus—mostly viruses. If all agents with a detection rate of > 10% were considered, norovirus, ETEC, astrovirus, and *Campylobacter* would also be included. This spectrum differed remarkably from that produced by the Global Enteric Multicenter Study (GEMS), which found rotavirus, *Cryptosporidium*, ETEC, and *Shigella* to be most common [23]. The most likely explanations for the discrepancy are the study design and differences in health-care access, economic development, and environmental conditions between our study sites and GEMS’ study sites.

Comparatively, bacterial pathogens may now play a less important role in children’s intestinal infection; more resources should be focused on viral infection in China. However, surveillance for bacterial pathogens must continue, since these bacteria are all foodborne pathogens and can rapidly cause large-scale outbreaks in certain circumstances [24]. A recent published meta-analysis indicated that abovementioned bacteria are distributed widely in food commodities in China [25].

ETEC is primarily associated with diarrhea in children < 5 years of age, as well as being a frequent cause of diarrhea in tourists visiting developing countries. EAEC was initially considered to be an opportunistic pathogen associated with diarrhea in immunocompromised patients or malnourished children [26]. Currently, EAEC is recognized as a cause of chronic diarrheal disease in children and adults in both developing and industrialized countries [27]. However, compared to ETEC, EAEC did not exhibit a prolonged course of illness in our study. Neither *E. coli* nor *Campylobacter* cause frequently observed infectious disease in China. The prevalence of these two bacteria in Chinese population is scarce, particularly among children. To date, just a few studies have been conducted in children < 5 years of age in Wuhan and Shanghai [28, 29]. However, the detection rates of EAEC, ETEC, and *Campylobacter* obtained from each study were inconsistent. The detection rates in Wuhan, a city in central China, were similar to those obtained in our study and were markedly higher than those in Shanghai, though conventional PCR methods, other than TAC assay were applied in Wuhan and Shanghai studies. These differences may be attributable to differences in sanitation and economic development between eastern and central regions. Notably, we observed, in a previous study, a statistical difference in prevalence of EAEC and ETEC between children with diarrhea and healthy controls, but no significant difference was observed in prevalence of *Campylobacter* [28]. This suggests that EAEC/ETEC may have greater public health significance than *Campylobacter* among Chinese children with diarrhea, and further research should focus on these pathogens. Shigellosis no longer appears to be a significant public health concern in China according to this study. The total number of cases infected with *shigella* in 2019 was 80,483, an incidence of 6.5 cases per 100,000 person-years [30].

In addition to study limitations noted elsewhere (5), we could not calculate odds ratios to confirm specific pathogens that caused diarrhea due to the absence of a healthy control group. In particular, subtypes of some microbial agents (such as *E. coli*, adenovirus, and enterovirus) may not be associated with diarrhea. Nevertheless, for the first time, our study provides a population-based, possible spectrum of pathogens for diarrhea among young children in China. Further verification of public health significance is planned to be performed through a birth cohort study being conducted in Zhengding, where a stool specimen is required from each participating child once a week regardless of their health status.

## Conclusion

Despite significant advances in fighting childhood diarrhea through sanitation improvement, safe water supplies, and vaccine development, diarrheal diseases remain one of the leading causes of childhood illness in China. Clearly, comprehensive understanding of prevalent etiologies is essential for the design and implementation of effective prevention strategies. Findings from our study support continued surveillance and attention for pathogens such as rotavirus and calicivirus that have gained increased awareness among the public and policymakers. More importantly, for neglected pathogens, such as EAEC, ETEC, *Campylobacter*, and non-enteric adenovirus, strengthened research should be performed to clarify epidemiological and clinical characteristics, and eventually confirm their significance for public health.

## Supplementary Information

**Additional file 1: Table S1.** Interrogated agents used in the TAC assay to test specimens from children with MSD in China.

## Data Availability

All the data supporting the findings are presented in the manuscript.
