# Supplementary material for: Burden and etiology of moderate and severe diarrhea in children less than 5 years of age living in north and south of China: Prospective, population-based surveillance
Source: Gut Pathog. 2021 May 24;13:33. doi: 10.1186/s13099-021-00428-2 (PMC8142869; doi:10.1186/s13099-021-00428-2)
Supplement: Supplementary file 1 — Additional file 1: Table S1. Interrogated agents used in the TAC assay to test specimens from children with MSD in China. [file 13099_2021_428_MOESM1_ESM.doc]

**Supplementary Table 1. Interrogated agents used in the TAC assay to test specimens from children with MSD in China.** For each sample, equal volumes (50 µl each) of extracted TNA were mixed with 50 µl qScriptTM XLT 1-Step RT-qPCR ToughMix® (VWR, Radnor, PA, USA). After thorough mixing, the reaction mixture (100 µl) was loaded into each port and centrifuged twice at 1200 rpm for 1 min. The card was then sealed and trimmed, and subsequent amplification and analysis were performed on the QuantStudio™ 7 Flex Real-Time PCR instrument (Applied Biosystems, Foster City, CA, USA). The cycling conditions were as follows: 45°C for 10 min and 94°C for 10 min, followed by 45 cycles of 94°C for 30 s and 60°C for 1 min.

| **Agents** |  | **Subgroups** |
| --- | --- | --- |
| Viruses | Adenovirus | 40/41 and others |
|  | Astrovirus |  |
|  | Enterovirus |  |
|  | Norovirus | GI and GII |
|  | Rotavirus | A, B, C and others |
|  | Sapovirus | I/II/IV and V |
| Bacteria | *Aeromonas* |  |
|  | *Campylobacter* | *coli* and *jejuni* |
|  | *Clostridium difficile* | *tcdA*, *tcdB*, and *paLOC* |
|  | Enteroaggregative *E. coli* (EAEC) | *aaiC* and *aatA* |
|  | Enteropathogenic *E. coli* (EPEC) | *eae* and *bfpA* |
|  | Enterotoxigenic *E. coli* (ETEC) | ST and LT |
|  | *Enterococcus* | *faecails* and *faecium* |
|  | *Mycobacterium tuberculosis* |  |
|  | *Salmonella spp* |  |
|  | Shiga toxin+ *E. coli* (STEC) | *stx1* and *stx2* |
|  | *Shigella*/enteroinvasive *E. coli* (EIEC) |  |
|  | *Vibrio cholera* |  |
|  | *Yersinia spp.* |  |
| Parasites | *Ascaris lumbricoides* |  |
|  | *Cryptosporidium parvum* |  |
|  | *Entamoeba histolytica* |  |
|  | *Giardia lamblia* |  |
|  | *Trichuris trichuria* |  |
